# 3D Finite Element Model on Drilling of CFRP with Numerical Optimization and Experimental Validation

**DOI:** 10.3390/ma14051161

**Published:** 2021-03-02

**Authors:** Patrick Hale, Eu-Gene Ng

**Affiliations:** Department of Mechanical Engineering, McMaster University, 1280 Main St. W, Hamilton, ON L8S 4L8, Canada; nge@mcmaster.ca

**Keywords:** CFRP drilling, cohesive surfaces, strain rate strengthening, innovative tool design

## Abstract

When drilling Carbon Fibre-Reinforced Plastic (CFRP) materials, achieving acceptable hole quality is challenging while balancing productivity and tool wear. Numerical models are important tools for the optimization of drilling CFRP materials in terms of material removal rate and hole quality. In this research, a macro-Finite Element (FE) model was developed to accurately predict the effect of drill tip geometry on hole entry and exit quality. The macro-mechanical material model was developed treating the Fiber-Reinforced Plastic (FRP) as an Equivalent Homogeneous Material (EHM). To reduce computational time, a numerical analysis was performed to investigate the influence of mass scaling, bulk viscosity, friction, strain rate strengthening, and cohesive surface modelling. A consideration must be made to minimize the dynamic effects in the FE prediction. The experimental work was carried out to investigate the effect of drill tip geometry on drilling forces and hole quality and to validate the FE results. The geometry of the drills used were either double-point angle or a “candle-stick” profile. The 3D drilling model accurately predicts the thrust force and hole quality generated by the two different drills. The results highlight the improvement in predicted results with the inclusion of cohesive surface modelling. The force signature profiles between the simulated and experimental results were similar. Furthermore, the difference between the predicted thrust force and those measured were less than 9%. When drilling with a double-angle drill tip, the inter-ply damage was reduced. This trend was observed in FE prediction.

## 1. Introduction

In aerospace and automotive industries, the interaction of different material components during assembly may require drilling holes to facilitate bolting sections together. Annually, 250 million twist drill bits are used in the US aerospace industry [1]. On the Airbus A350, it is estimated that 55,000 holes are drilled to facilitate the assembly of one unit [2]. Composite plates with holes that have been moulded or drilled are susceptible to damage. Zitoune et al. demonstrated by loading parts under tension that parts with drilled holes results in a 30% decrease in fracture strength [3]. Moulded holes are not always feasible, and attaining positional and size tolerances becomes more cumbersome versus drilling, thereby creating motivation to improve the drilling process.

Contrary to the shear-based cutting mechanism in ductile metals, CFRPs are dominated by a brittle crack propagation [4,5]. In CFRPs, the high thrust force resulting from the drill can cause a peel-up and push-out effect on the workpiece, resulting in delamination of the ply. As drilling initiates, the work material resists the thrust force induced by the chisel edge of the tool. As the drill approaches the exit surface, there is little material to withstand this thrust force. Therefore, a significant thrust force is transferred to the interface between the plies, causing delamination under pure bending. By identifying the critical thrust force causing delamination with respect to uncut thickness of the laminate, the feed rate should be modified throughout the progression of the drilling. Mainly, an aggressive feed at the hole entrance to promote the Material Removal Rate (MRR) and a reduced feed to mitigate delamination near the exit of the cut [6]. Adding to these considerations, tool wear increases the dynamic nature of the drilling process. Ismail et al. described the unavoidable phenomenon, which requires coolants, tool life monitoring and prediction, effective drill design geometry, and optimal process parameters [7].

Vijayaraghvan outlines numerous considerations in modelling multilayer material machining, which includes material modelling, contact, fracture criteria, adaptive meshing, element types, and tool modelling [8]. Significant work was done to investigate the drilling of CFRP materials, including reviews completed by Panchagnula et al. [9] and Lissek et al. [10], outlining the significance of process monitoring to ensure hole quality. Kahwash et al. highlighted the current practice of modelling the cutting process of CFRPs and the use of 2D orthogonal cutting due to its simplicity and computational advantage [11]. Liu et al. from experimental results on drilling of composite laminates concluded that the variation between materials’ elastic modulus affected the drilled hole diameters [12]. Computationally, Mahdi et al. studied mesh sensitivity, plane stress versus strain, and rake angle. This research concluded that the rake angle had a minimal effect. The effect of fibre angle when machining FRPs was successfully demonstrated [13]. Shyha et al. studied the effect of machining process parameters when drilling CFRPs and determined that drill geometry and feed rate were the most critical [14]. Faraz et al. studied the effect of cutting edge rounding to predict and prevent increase drill loads and to maintain hole quality [2].

Arola and Ramulu produced preliminary 2D orthogonal cutting models of graphite epoxy material and attained a good correlation between predicted and experimental cutting force. However, poor agreement was found with thrust force [15]. An orthogonal 3D cutting model of CFRP was published by He et al. [16]. Strong variance in predicted cutting forces was described when using different failure criteria including Hashin and Max Stress. Max Stress predicted cutting force reasonably where Hashin underestimated significantly. Predicted thrust force was significantly underpredicted by a 75% difference when compared to experiments. He et al. described the thrust force predictions as an order of magnitude less than experiments as found in literature [17,18]. No element removal or chip formation was captured. Lasri et al. described the magnitude of difference between how experimental and modelled thrust force captures damage but did not show the removal of elements and chip formation progression, only the initiation [18].

Phadnis et al. compared drilling experiments to FEM model. Using X-ray micro-tomography, drill entry and exit delamination damage were investigated [19]. Although exaggerated, the outer region of the delamination damage predicted in the Finite Element Analysis (FEA) resembled the experiments. The concern is the significant element removal of the failed cohesive elements at exit. Jain et al. [20], Lissek et al [10], and Won et al. [21] investigated the relationship between thrust force on hole quality with respect to delamination damage. Jain et al. discovered reduced thrust force along with delamination damage when the chisel width edge of the drill was decreased. Won el. al. noticed reduced thrust force measurements by pre-drilling the holes first. The results concluded that the magnitude of the thrust force critically affects the delamination damage of the CFRP.

The progression of damage experienced by an element is influenced by the cutting speed. The stable time increment in a FE analysis is governed by element size, bulk modulus, and material density. The fine mesh and high stiffness of CFRP develops rigid elements that propagate a stress front through the CFRP, creating premature damage. The bulk viscosity parameter can more accurately represent the material response by facilitating dampening; otherwise, it is not well represented in the model. Garekani described a FE model convergence sensitivity due to excessively distorted elements at certain cutting speeds. At high cutting speeds, models progress further before encountering convergence issues. When the cutting speed is reduced, a limited region exists where elements’ material properties have been successfully degraded but the damage is not fully saturated and, therefore, elements cannot be removed [22]. For damage to be fully saturated, the longitudinal damage must be satisfied, which is not always possible depending on the loading. Garekani suggests a max degradation parameter control less than unity to prevent this convergence issue. This facilitates convergence but does improve the material representation. Bulk viscosity parameters are frequently stated [23], with little description to why and how linear and quadratic viscosity parameters are determined or their influence on the model.

Conventional drilling experiments investigating cutting speed and CFRP constituents outlining significant importance of the matrix on the material response due to strain rate and thermal effects were studied by Merino-Perez et al. [24]. Merino-Perez et al. described a decline in the matrix ability to properly transfer load between fibres at high strain rates. Lasri et al. described minimal strain rate dependence and a bouncing back effect reducing the overall depth of cut with respect to the fibre material [18]. Using the split Hopkins bar technique, Lifshitz experimentally studied the interlaminar failure of CFRP at strain rates of 100–250 s${}^{-1}$ versus at static test conditions. The results showed an increase in strength by an average of 36% and modulus by 30% at the higher strain rate [25].

Giasin et al. created a 3D drilling model of a hybrid material made of stacked glass fibre/epoxy prepreg with aluminum sheets. The model predicted torque within 0.83–17.9% and thrust force within 3.2–53.2%. However, no delamination was identified and was deemed negligible [26]. The effect of including a cohesive surface in finite element modelling on the cutting of CFRP was investigated by Dandekar et.al. [27], Abena et al. [28], and Lasri et al. [18], among others. Lasri et al. used a progressive failure stiffness degradation scheme when modelling orthogonal cutting to gain an understanding on subsurface damage and its contribution to chip formation [18]. The inclusion of an interface zone between the constituents was proven to have significant effect on delamination magnitude and fibre/matrix failure. Three-dimensional orthogonal and drilling model produced by Usui et al. captured delamination in various fibre orientations by using cohesive zone elements mapped to fracture planes defined by the Miller indices [29]. Zenia et al. developed similar research with an elastoplastic damage model VUMAT that predicts interply damage and chip formation [30]. Isbilir et al. modelled the drilling process, including the inter-laminar damage, comparing a standard twist drill and step drill geometries [31]. Ply damage was modelled using Hashin’s theory, and delamination was based on a cohesive contact relationship. Isbilir et al. described better model prediction capabilities with the inclusion of inter-laminar cohesive modelling.

The objective of this work was to progress the development of a macro FE drilling model to be used as a tool to accurately test various drill geometries with reasonable computation time. To substantially reduce computational time, numerical analysis was preformed to investigate the influence of mass scaling, bulk viscosity, friction, strain rate strengthening, and cohesive surface modelling, building on intra-ply and inter-ply progressive failure modelling techniques developed in [32,33]. Experimental work was carried out to investigate the effect of of drill tip geometries on drilling forces and hole quality and to validate FE model prediction capabilities.

## 2. Experimental Work

The pre-impregnated unidirectional CFRP panels were procured from ACP Composites Inc. using an autoclave curing process. The mechanical properties described by ACP Composites Inc. [34] are tabulated in Table 1.

**Figure 1:**
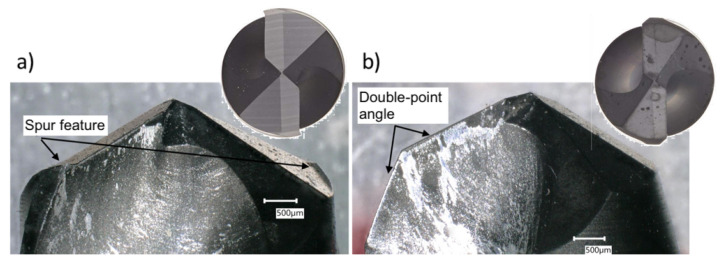
Drill geometries (**a**) CD854 and (**b**) CD856.

Two drill geometries were tested experimentally, named the CoroDrill (CD) CD854 and CD856, and are shown in Figure 1a,b, respectively, after the drilling experiments were completed (Sandvik, Gimo, Sweden). The CD856 has a double-point angle, carbide geometry with diamond coating (N${}_{2}$OC) that is designed to reduce delamination and splintering. The diamond coated (N${}_{2}$OC) CD854 has a point geometry with additional spur edges on the circumference, designed to minimize burr formation when drilling aluminum, detailed in Figure 1a. Both CD drills have a diameter of 4.7625 mm and incorporate small point angles (CD854—130°, CD856—120°) and high rake angles to reduce axial forces, critical for drilling thin walled structures [35]. Drill geometries were inspected with digital microscope, measuring key features from output frames. The CD854 drill incorporates additional spurs on the perimeter of the tool that score the circumference of the hole, as detailed in Figure 1a. This design was inspired by a Brad-Point drill commonly used in wood working to prevent fibre pull and tearing of the wood.

The drill tests were carried out on a Fanuc controlled Matsuura LX-1, 3-axis vertical CNC machine (Matsuura Machinery Corporation, Fukui, Japan). Cutting forces were measured using a Kistler three-component stationary piezoelectric dynamometer (type 9272, calibrated range: Fx = 0–3000 N, Fy = 0–3000 N, and Fz = 0–6000 N), connected to a series of charge amplifiers (type 5011A) (Kistler Group, Winterthur, Switzerland). Data acquisition was accomplished using an analogue-to-digital converter card connected to a high-performance computer, which was capable of sampling at 200 K samples per second per channel. Recordings were measured at 10 kHz per channel. The drilling process was repeated five times to ensure repeatability. The machining parameters employed were 0.05 mm/rev and 60 m/min as recommended by the tool manufacturer.

## 3. Finite Element Modelling Considerations

A macro-mechanical approach treating the FRP as an Equivalent Homogeneous Material (EHM) was developed. The EHM model is based on Multi-Continuum Theory (MCT), which used a Representative Volume Element (RVE) to express the composite stress state, as shown in Equation (1) [36],

(1) $$\sigma^{c}=\frac{1}{V}\int_{D}\sigma \left(x,y,z\right)dV$$

where *V* is the total RVE and *D* is the fibre-matrix domain. The fibre and matrix average stress states are shown in Equations (2) and (3), respectively [36]:

(2) $$\sigma^{f}=\frac{1}{V_{f}}\int_{D_{f}}\sigma \left(x,y,z\right)dV$$

(3) $$\sigma^{m}=\frac{1}{V_{m}}\int_{D_{m}}\sigma \left(x,y,z\right)dV$$

where $V_{f}$ if the fibre RVE and $V_{m}$ is the matrix RVE. The constituent average stress and strain for the fibre and matrix ($\sigma^{f},\sigma^{m},$$\epsilon^{f},\epsilon^{m}$) are critical to predict damage and material failure versus a homogenized average stress and strain ($\sigma^{c},\epsilon^{c}$) [36]. When a FRP is subject to non-parallel loading with respect to the fibre orientation, failure is dominated by the matrix constituent. Damage should not be controlled by the fibre, and the matrix damage should not act only as a contribution to the homogenized composite material failure. This is a significant limitation of prior EHM modelling versus the MCT-EHM formulation. If a failure criterion is independent and based only on a matrix parameter, it can initiate element deletion.

FE modelling of Fibre-Reinforced Polymers (FRPs) forms its basis on the failure model developed by Hashin [37] that encompasses four failure modes, which are (i) matrix tension, (ii) matrix compression, (iii) fibre tension, and (iv) fibre compression failure. Significant work has developed numerous additional failure models including LaRC02 [38,39], Max Strain, Max Stress, Tsai-Wu [40], Tsai-Hill [41], Christensen [42], Puck et al. [43], and a Multi-Continuum Theory (MCT) [44] failure criterion. The formulation and advantages of these failure models are described in previous literature [32,33]. In this research, a user-defined material subroutine was implemented into ABAQUS to utilize failure criteria alternative to the built-in Hashin method for FRPs.

When damage is initiated, controlled by the failure model implemented, an instantaneous degradation method reduces the stiffness of the matrix and fibre from its original undamaged state to a user-defined value between zero and one. This degradation scheme is implemented instantaneously or can be defined for a time period to improve the response prediction of fracture. This is an efficient degradation method; however, it can be sensitive to mesh size, resulting in increased failure loads for coarse meshes and premature failure for refined mesh models. To avoid premature failure prediction of the CFRP loading response, techniques including damping applied through bulk viscosity, softening in contact interactions, and enhanced element controls reducing stiffness can be applied.

### 3.1. Finite Element Geometry and Boundary Conditions

The drilling FE model was designed to replicate the experimental setup using a rigid drill bit. Drill bits were designed in Siemens NX CAD, exported as step (.stp) files, and imported into ABAQUS. The complex drill geometries require the use of tetrahedral 3D stress elements (C3D10M). The C3D10M are explicit, quadratic, modified elements with the deformation along the edge following a bi-linear interpolation versus a quadratic function. This modification creates an additional node in the middle of the edge of the element. The modified term refers to a unique formulation using a bi-linear interpolation. These elements cannot represent curved surfaces as well as the true second-order elements but gain computational efficiency [45]. Cyclic symmetry is not possible in ABAQUS Explicit models. As the tool revolves about the z-axis, the interaction between the tool and workpiece would be cyclically symmetric except for the changing interaction between the tool face and fibre orientation. Symmetry in the XZ and YZ plane was utilized to reduce the workpiece to one quarter and therefore reduced the computation time but still captured a tool–fibre interaction ranging from 0 to 90°.

**Figure 2:**
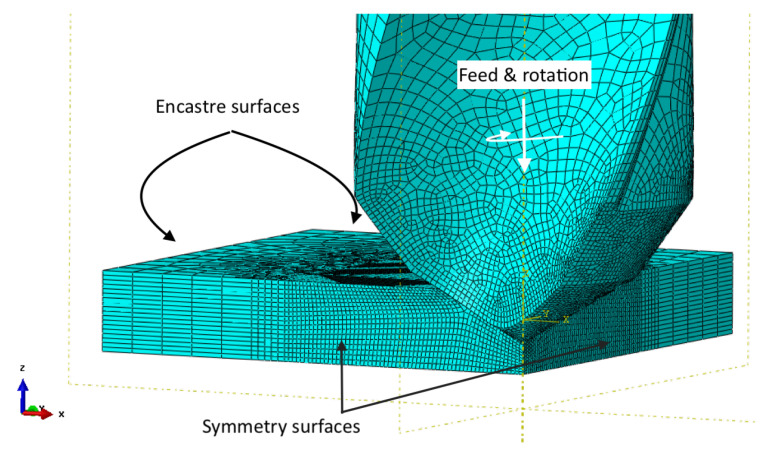
Model boundary conditions illustration.

The body elements and nodes for the drill are mapped to the reference point; load constraints are most efficiently applied with this setup. When drilling CFRPs, the loading velocity in the z-axis direction is 3.342 mm/s (feed = 0.05 mm/rev) and rotation is 420.0 rad/s (4010 RPM, 60 m/min cutting speed). The model boundary conditions are shown in Figure 2. The feed rate and cutting speed magnitudes are recommended by the tool manufacturer for the CoroDrill CD854 and CD856 drills [46].

**Figure 3:**
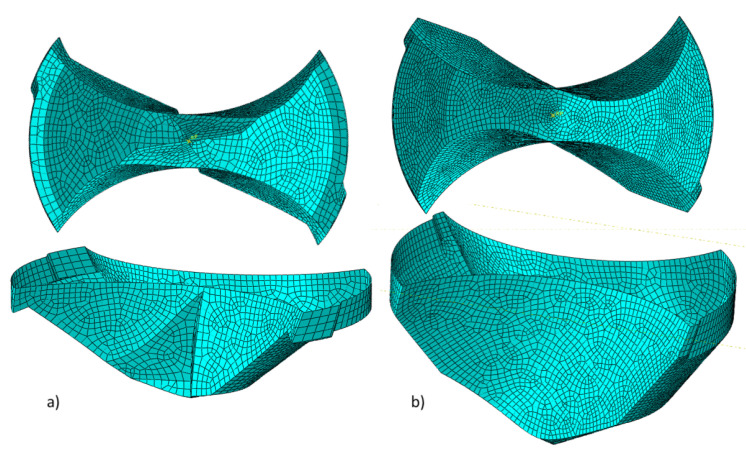
Discrete rigid (shell) body: (**a**) CD854 and (**b**) CD856.

A discrete rigid body converts the solid geometry to a shell body. The tip of the drill bit is shown in Figure 3. This negates the requirement of meshing the internal volume and focuses on the outer surface using a 4-node bi-linear rigid quadrilateral element (R3D4). This setup requires additional property assignment including the geometries’ weight and rotational inertia. The approach reduced the number of elements from 67,672 to only 6568 elements using the discrete rigid geometry.

The CFRP laminate workpiece was modelled as one 3D deformable member. The sample is subdivided with 10 plies to facilitate cohesive surface interactions. The material properties for the CFRP were determined and validated through tensile and three-point bending experiments and FE models at various fibre orientations [32,33].

In the explicit analysis, the stable time increment decreases as Young’s Modulus increases [45]. The tool in a machining simulation that is generally quite stiff and rigid compared to a workpiece can negatively affect the stable time increment. A discrete rigid body does not affect the global time increment and, therefore, can increase computational efficiency without significantly affecting the overall accuracy of the solution. Comparing identical models except for the described differences in the drill bit representation, the explicit time step for a deformable body with a rigid body constraint is 1.22 × $10^{-9}$ s but that for a discrete rigid body is 3.43 × $10^{-9}$ s. An increase in stable time increment of three times and reduction in computation time is observed. The estimated memory required for the otherwise equivalent analyses reduces from 1.9 GB to 1.1 GB due to the reduced elements required for meshing the tool. The resultant force prediction is subjected to noise. The noise was generated by the high modulus of CFRP under high strain rate deformation together with element deletion, leading to intermittent contact, and small modelling volume, which provide minimal damping. Therefore, the resultant force is filtered with a Chebyshev type II filter.

### 3.2. Mass Scaling

The minimum stable time increment for an explicit dynamic analysis is expressed in Equation (4) [45],

(4) $$\Delta t=L^{e}/c_{d}\;,\;\;\;and\;\;c_{d}=\sqrt{\frac{E}{\rho }}$$

where $L^{e}$ is the characteristic length element and $c_{d}$ is the dilatational wave speed of the material. The dilatational wave speed requires Young’s modulus (*E*) and the density (ρ) of the material [45].

With a characteristic element length of 10–100 μm, the stable time increment is approximately 1.0 × $10^{-8}$ s to 1.0 × $10^{-9}$ s, resulting in an extremely long model computation time involving 1.0 × $10^{6}$ s to 10 × $10^{7}$ s solution increments. To improve computational efficiency with explicit dynamic models, scaling techniques can be employed via time scaling or mass scaling. The dynamic effects induced by changing the loading time or inertial effects resulting from increased density to increase the time increment must remain insignificant. Mass scaling is achieved by increasing the density of the smallest elements, which modifies the wave speed and the resulting time step.

**Figure 4:**
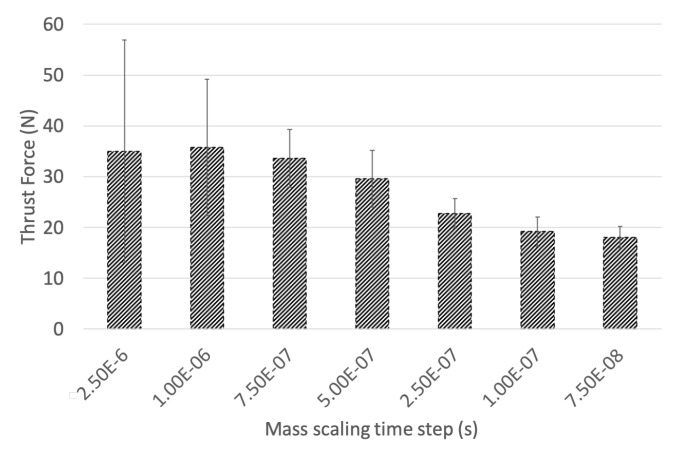
Effect of mass scaling on thrust force.

Table 2 compares mass scaled models controlled with a time step increment ranging from 2.5 × $10^{-6}$ s to no scaling. Without scaling, the model would compute in 175 days. The mass in the system without scaling is 0.169 kg and increases to ≈6 kg at the most extreme scaling studied. Figure 4 compares the resulting thrust force with mass scaling controlled by the time step, on the range from 2.5 × $10^{-7}$ s to 7.5 × $10^{-8}$ s. A large oscillation response is generated by the increased mass in the model when heavily scaled, resulting in exaggerating the thrust force impact in the CFRP workpiece. The range bar in Figure 4 illustrates the oscillation magnitude. This produces a shock wave into the workpiece, with an exaggerated damage front.

The 7.5 × $10^{-8}$ s time step results in a mass scaling of 2.91% and develops a smooth progression through the loading profile. However, this time step requires significant computational resources. The 1.0 × $10^{-7}$ s time step involving 5.30% scaling reduces the computational burden by 3 days while avoiding a harmonically induced thrust force loading response. The time step of 2.5 × $10^{-7}$ s was used to investigate the effect of model input parameters on model response, seen in Section 3.3, Section 3.4, Section 3.5, Section 3.6. For the 3D drilling model, a time step of 1.0 × $10^{-7}$ s was used.

### 3.3. Bulk Viscosity

When modelling high-rate, dynamic situations, bulk viscosity applies damping with respect to the volumetric straining [45]. In an explicit analysis, the bulk viscosity is applied in a linear and quadratic form. The linear bulk viscosity is used to damp the resonant in the highest element frequency and is expressed in Equation (5) [45],

(5) $$p_{1}=b_{1}\rho c_{d}L^{e}\dot{\epsilon_{vol}}$$

where $b_{1}$ is the damping coefficient that defaults to 0.06 and $\dot{\epsilon_{vol}}$ is the volumetric strain rate. The quadratic bulk viscosity is used to distribute the shock front from a compressive load and prevent elements collapsing from the high velocity gradient and takes the form as shown in Equation (6) [45],

(6) $$p_{2}=\rho {\left(b_{2}L^{e}\dot{\epsilon_{vol}}\right)}^{2}$$

where $b_{2}$ is the quadratic bulk viscosity coefficient with a default variable of 1.2 [45].

To understand and optimize the effect that linear and quadratic bulk viscosity have on the drilling simulation, a parametric study was carried out to investigate the effect of these parameters at two magnitudes and is tabulated in Table 3.

**Figure 5:**
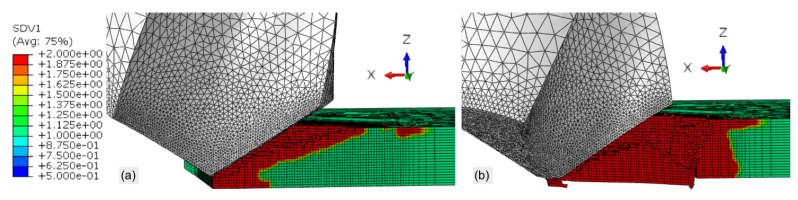
(**a**) Pre- and (**b**) post-bulk viscosity induced damage when $b_{1}$ = 1.1 and $b_{2}$ = 2.5.

Phase I outlines the significant impact that the bulk viscosity parameters have on the drilling simulation. In comparison to the default magnitudes (0.06 and 1.2), the predicted thrust force when the linear and quadratic viscosity parameters are set to 1.1 and 2.5 is four times greater. The mass scaling for the model increases by a factor of 4.92 to maintain the time step of the analysis, affecting the load response of the tool. Figure 5 illustrates pre- and post-bulk viscosity damage induced into the workpiece. This is an unrealistic response demonstrating poor prediction capabilities due to incorrect, excessive bulk viscosity parameters. Shown in Figure 5b, the majority of material beneath the tool fractures cause a spike in the load output not observed with experiments.

**Figure 6:**
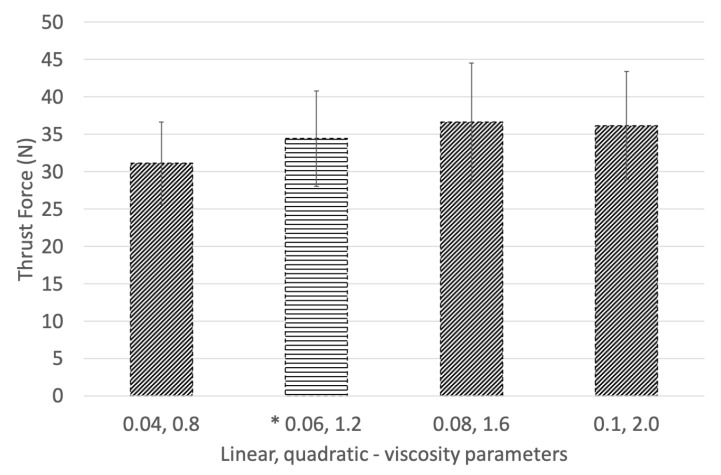
Effect of linear and quadratic bulk viscosity on simulated thrust force (* Default parameters).

In phase II, the bulk viscosity parameters were modified more closely with respect to the default parameters of 0.06 (linear) and 1.2 (quadratic). Figure 6 illustrates the thrust force for the phase II viscosity parameters studied. Case “0.04, 0.8” did not provide enough dampening to mitigate the oscillating response present in the loading profile. The default parameters develop a profile inclusive of a loading region with a constant tool engagement region, followed by a decrease in loading as the tool exits, as observed in the experiments. An increase from the default parameters to “0.08, 1.6” predicts a similar response while reducing the oscillation output. The case “0.1, 2.0” most effectively develops the loading response observed experimentally, with the least oscillation in the predicted thrust force.

### 3.4. Friction

Friction opposes motion between surfaces in contact. As failed elements are removed from the modelled workpiece while drilling, new elements are exposed. An interior node set creation facilitates contact between the tool and the newly exposed elements. Contact in an explicit FE model involves a general contact regime that can be enhanced identifying surface pairs.

Normal behaviour between the surfaces applies a “hard” contact to prevent pressure-overclosure. The tangential behaviour applies a penalty friction formulation isotropically. Chardon et al. highlighted the large variety in literature regarding the friction coefficient when machining CFRP, noting researchers using values ranging from 0.09 to 0.9 [47]. The penalty formulation uses Coulomb’s friction relationship that determines the maximum allowable shear stress across an interface as a function of the contact pressure. Once the magnitude of the shear stress surpasses the stick/slip point, the contacting surfaces slide [45]. Coulomb’s friction relationship is given in Equation (7),

(7) $$F_{R}=\mu F_{N}$$

where μ is the coefficient of friction and $F_{N}$ is the normal force. Different coefficients of friction for static and kinetic contact exist, resulting in different static and kinetic friction forces. Neither static nor kinetic frictions demonstrate high dependence on contact area between surfaces or roughness but rather the pairing of materials [48].

**Figure 7:**
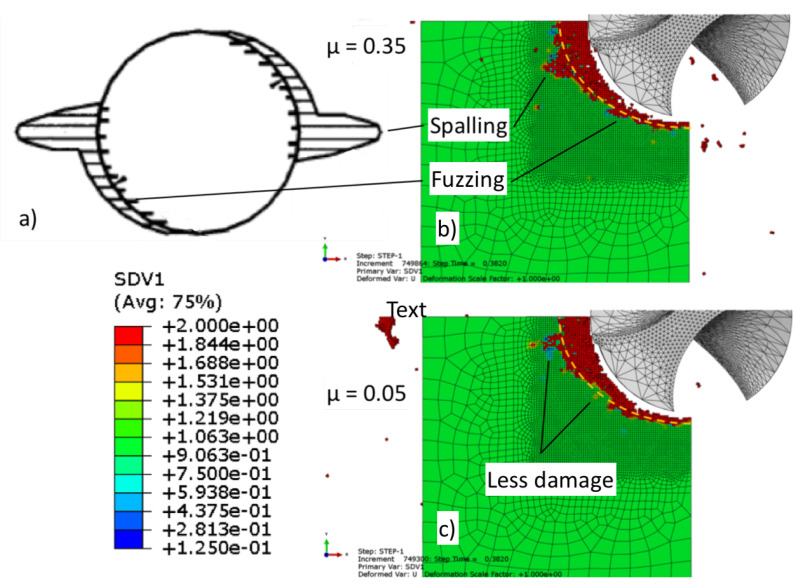
(**a**) Illustration of drilling-induced damage by fuzzing and spalling [49] and SDV1 damage distribution at (**b**) 0.35 and (**c**) 0.05 coefficients of friction.

Figure 7a illustrates common damage experienced when drilling UD-CFRP including fuzzing and spalling [49]. Fuzzing refers to uncut fibres around the hole that develop when the angle between the fibre and the cutting velocity are acute. Spalling damage is a form of delamination resulting from the chisel-edge of the drill and develops further as a result of the cutting edges on the side of the drill [49]. Figure 7 illustrates the fibre damage (SDV1) output comparing friction coefficients (b) 0.35 and (c) 0.05. The damage observed in the model increased when a greater coefficient of friction was used. Shown in Figure 7b,c, using an identical arc for reference, the increased damage in addition to areas of fuzzing and spalling is noticeable in (b) when the greater friction coefficient is used.

**Figure 8:**
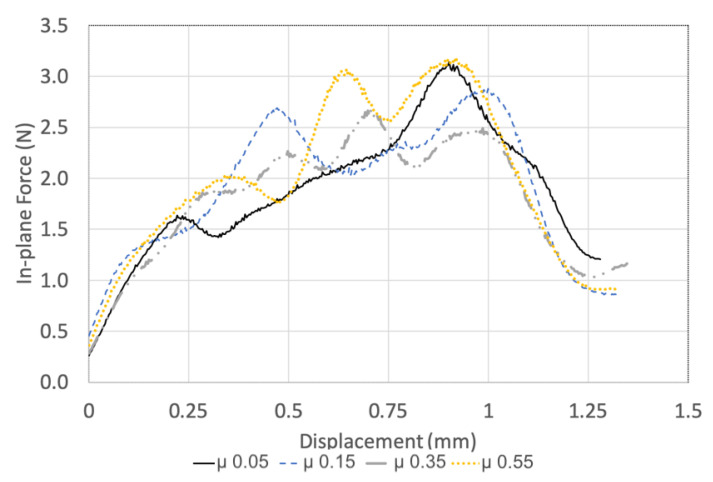
Effect of coefficient of friction on in-plane force displacement profile.

The effect of the friction coefficient on the FE drilling model thrust force and in-plane force is tabulated in Table 4. The magnitude of the predicted thrust force is not significantly influenced by the friction coefficient. Notable differences in the in-plane force–displacement profile are observed, shown in Figure 8, despite minimal change in magnitude, as shown in Table 4. When the friction coefficient is 0.55, an unsteady oscillating load response develops and an increased in-plane force is observed. The 0.05 friction coefficient does not suffer an oscillating response; however, a load spike is observed at the drilling exit. Modifying the friction coefficient changes the interaction between the tool and UD-CFRP. This influences the damage and force prediction; however, no discernible, quantifiable relationship was determined. Prakash et al. studied friction coefficients from 0 to 1, noting the magnitude that most closely represents the experiments [50]. Rather than fitting the model, Chardon et al. performed experiments to capture the tribological conditions when machining CFRP and described an apparent friction coefficient of 0.06 to 0.08 [47]. The drilling models in this research demonstrate improved prediction with low-magnitude coefficients of friction agreeing well with [47]. Therefore, a friction coefficient of 0.1 was used in the drilling models.

### 3.5. Strain Rate Strengthening

In drilling operations, the strain rate can be 1000 s${}^{-1}$, and therefore, consideration should be made for the strain rate hardening experienced by the epoxy in a CFRP laminate. This is accomplished by invoking two additional user material variables in the VUMAT and are based on the relationship in Equation (8) [36]:

(8) $$S=S_{0}\left(1+\zeta_{m}log_{10}\frac{\dot{\epsilon }}{\dot{\epsilon_{0}}}\right)$$

where $s_{0}$ is the strength at a strain rate of $\dot{\epsilon_{0}}=0.001$ $s^{-1}$, $\zeta_{m}$ is the user material magnitude for matrix strain hardening. For reference, a matrix strength of 50 MPa and a $\zeta_{m}$ of 0.1 resulted in a strength of 75 MPa when the strain rate ($\dot{\epsilon }$) is 100 s${}^{-1}$. When the strain rate is increased to 1000 s${}^{-1}$, the matrix strength becomes 80 MPa based on this relationship.

**Figure 9:**
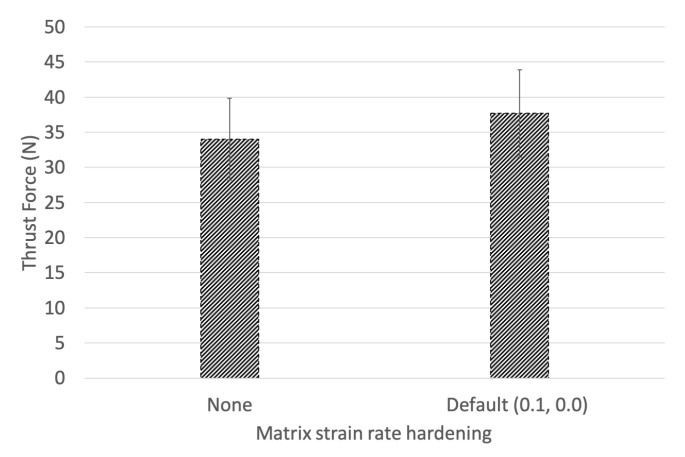
Matrix strain rate hardening.

A sensitivity analysis regarding the matrix strain rate strengthening parameter was investigated. A model without matrix strain rate strengthening was compared against models setting the strengthening parameters to 0.01, 0.3, 0.5, and 0.99. Figure 9 shows the thrust force magnitude with and without strain rate strengthening of the matrix plotted. The strain rate hardening parameter increases the loading response by 10.87%. The load–displacement response was insensitive to the magnitude of the strain hardening parameter (0.01 to 0.99) despite the relationship described in Equation (8). All models studied with the inclusion of the strengthening relationship output the same response. Although the sensitivity analysis was not conclusive, the 10.87% increase in thrust force prediction is in line with the experimental findings described by Lifshitz et al. [25]. Lifshitz et al. described a sensitivity of 1–3 times increase in modulus depending on the loading arrangement with respect to fibre orientation. Although many authors describe the fibre materials’ insensitivity to strain rate [15,17,18], the matrix and inter-ply interaction demonstrates strain rate sensitivity and directly influences the transfer of load between the fibre and matrix [24].

### 3.6. Cohesive Surface Modelling

When a UD-CFRP laminate is loaded in a principal direction such as tension, compression, or bending, cohesive surface modelling is a useful mechanism that can facilitate more accurate prediction of the nonlinearity in the CFRP material response. A linear-elastic fibre material that follows a damage-degradation process cannot sufficiently capture this, reiterating the vitality of cohesive surfaces [32]. Incorporating nonlinear material response prediction capabilities is seldom attempted in research due to the added modelling complexity and computational expense, despite experimental testing demonstrating its effect [51,52].

To model, the delamination damage when drilling a CFRP laminate, the geometry was divided into individual instances with cohesive surface interactions applied between them. The CFRP workpiece has a thickness of 1.1 mm and was divided into 10 sections to represent the ply thickness. Node sets are created for the top and bottom surfaces to be used with the cohesive interactions. Individual property assignments are created for the cohesive surface interactions based on prior experiments; details are described in [32,33].

A cohesive contact is modelled using normal behaviour, cohesive behaviour, and damage behaviour properties. The damage modelling parameters are the most influential regarding the response of the cohesive surface interaction. This involves identifying a damage initiation point and controlling the damage evolution. Damage evolution is initiated, where the stiffness of the cohesive surface will be degraded. Failure separation criteria was controlled by an effective separation at complete failure, relative to the initiation of damage as discovered by [36,38,45]. Damage stabilization was applied.

**Figure 10:**
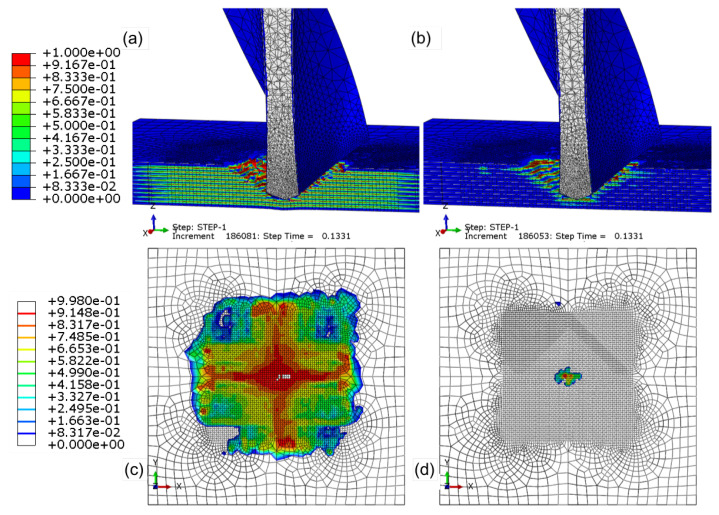
Cohesive damage when using MAXS damage initiation at (**a**) 70 MPa and (**b**) 250 MPa. Cohesive damage between plies 9 and 10 (**c**) 70 MPa and (**d**) 250 MPa.

Cohesive modelling between plies is an additional consideration that was implemented to predict the peel-up and break-out damage when drilling UD-CFRP laminates with various drill geometries. This damage has been shown to be most critical to determine a parts functionality [49,53]. Figure 10a,b details cohesive surface modelling that has damage initiation values based on the in-plane shear strength of 70 MPa and the transverse (90°) compressive strength of the CFRP of 250 MPa, respectively. The damage initiation variable ranges from 0, representing no damage, to 1, representing that damage initiation is complete and that damage evolution is initiated. If damage is initiated in the cohesive surface at an inaccurate, lesser strength, the damage will propagate too drastically between the plies, as shown in Figure 10c. As the cohesive damage increases, the laminate will become multiple, un-bonded plies that are substantially weaker than the original laminate, leading to laminate failure at a lesser strength. The damage initiation and evolution parameters must accurately predict the cohesive relationship. Otherwise, unrealistic damage will result in poor model prediction capabilities, which was also concluded by [19]. Shown in Figure 10b,d, the 250 MPa cohesive surface damage initiation strength accurately predicts the damage induced by the thrust and rotation of the drill. Made clear in the figure is the importance of the cohesive bond between the stacking of the plies, which must resist the compressive, bending load induced by the drill and the shearing of the cohesive bond due to the rotation and therefore cutting of the tool.

## 4. Results and Discussion

**Figure 11:**
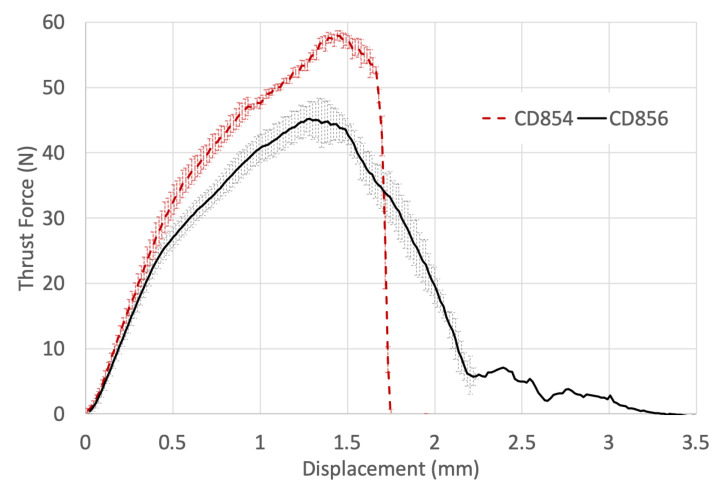
Experimentally measured thrust force signature of CD854 and CD856 versus drill displacement.

Figure 11 shows the thrust force with respect to the drill displacement measured experimentally when drilling CFRP with CD854 and CD856 drill bits. There is an increased thrust force of 22.3% experienced for the CD854 but a more immediate exit of the workpiece in comparison to the CD856. The immediate exit was observed as the thrust force signature became zero at a drill displacement of 1.75 mm due to the shallower axial drill tip geometry. The more immediate exit of CD854 drill from the CFRP laminate is due to the spur features that are positioned at the forefront of the drill axially and located at the periphery of the tool, radially. As these spurs exit the laminate in the axial direction, the majority of the hole has been cut radially. In contrast, the CD856 double-point angle results in a delayed, reducing load as it completes the drilling through the CFRP. The tool must travel deeper axially to allow for the remainder of the diameter to be machined. This results in the CD856 double-point angle design experiencing a lower thrust force and agrees well with thrust profiles determined in the research done by Li et al., who determined lower thrust and improved hole quality in comparison to standard twist drill geometries [54].

**Figure 12:**
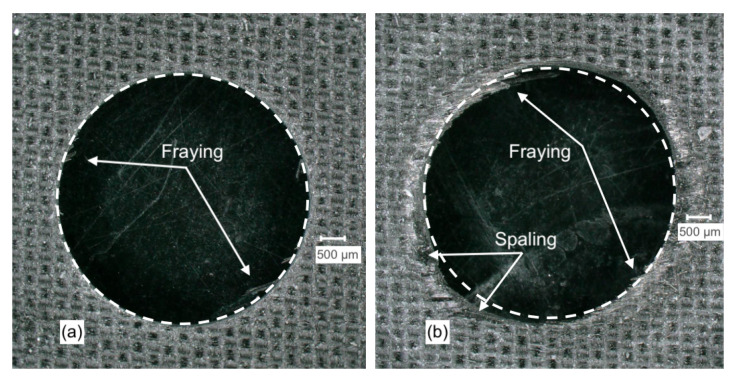
Hole entry quality when drilling with (**a**) CD854 and (**b**) CD856.

Figure 12a,b shows the hole entry quality with CD854 and CD856 drills, respectively. When drilling with CD854, the amount of fraying at the hole entry was noticeably minimized in comparison to the CD856. Geometrically, a more circular hole was generated with the CD854 versus the CD856. This is evident when the identical white-dashed reference circles were superimposed on the top of the drilled holes in Figure 12a,b.

**Figure 13:**
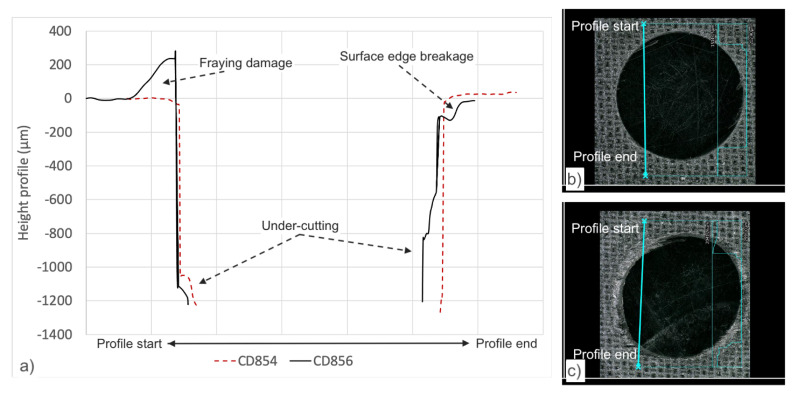
(**a**) Depth profile of the hole drilled with CD854 and CD856 drills, (**b**) visual of hole entry quality when drilling with the CD854 drill, (**c**) visual of hole entry quality when drilling with the CD856 drill.

Figure 13 details a visual description of the hole entry drilled with CD854 and for CD856. Significant fraying was observed at the hole entry when drilling with CD856 as observed in Figure 13c. Xu et al. studied double-point angle drill geometries and described similar fraying and tearing defects, as shown in Figure 13b [55]. Figure 13a details the depth profile of the holes. It was observed when drilling with CD856 that undesired under-cutting of the hole wall is reduced when compared to the CD854 drill. Qui et al. also described increased fraying and burrs with the double-point angle drill versus the improved hole quality with candle-stick geometry [56].

**Figure 14:**
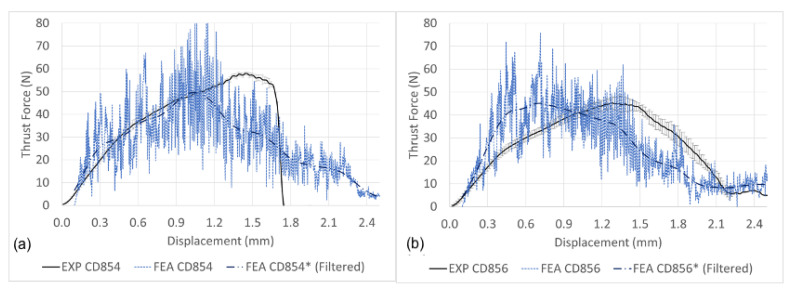
Experimental and predicted thrust force: (**a**) CD854 and (**b**) CD856.

Figure 14a,b details the thrust force signature with respect to depth drilled with CD854 and CD856 drills, respectively. The difference between the predicted and simulated maximum thrust force for CD854 and CD856 were 6.58% and 0.39%, respectively. The modelled thrust force signature for CD854 was unable to predict the rapid decrease when the drill exits the CFRP. Instead, a slower rate of decrease was shown. On the other hand, the predicted thrust force signature for the CD856 is similar to those acquired experimentally in both the drilling initiation and exit of the holes, as shown in Figure 14b.

**Figure 15:**
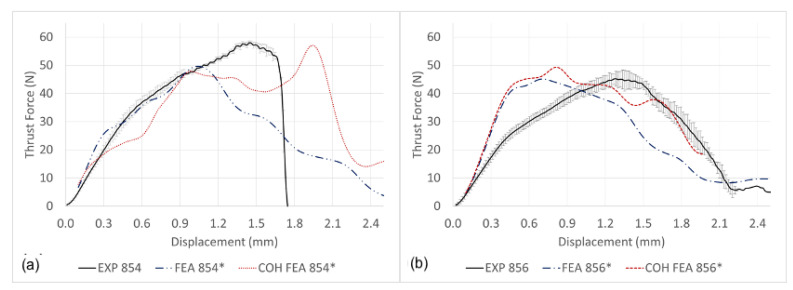
Experimental and predicted thrust force signatures with cohesive (COH) surface modelling: (**a**) CD854 and (**b**) CD856.

The thrust force signature prediction excluding and incorporating cohesive surface modelling together with experimental results is shown in Figure 15a,b when drilling with CD854 and CD856, respectively. Shown in Figure 15a,b, the cohesive surfaces provide additional strength and dampening, preventing the compressive shock-wave from prematurely causing elements to fail in the FE model. As a result, the thrust output predicted a more realistic steady cutting zone before more realistically exiting the CFRP laminate. When the cohesive surface modelling was not considered in the simulation, this was not observed, reiterating the significance of incorporating the cohesive interaction in FE models. Effective cohesive surface modelling makes the observation of damage between plies possible and develops a more realistic material representation. The thrust profile prediction with cohesive surface interactions demonstrates better prediction in comparison to the experiments. Karprat et al. who studied various double-point angle drill geometries described similar observations [57,58]. The cohesive surfaces develop a load profile that extends over a greater drilling displacement, reducing the amount of prematurely failed elements and more accurately predicts the experimental results. In comparison to the drilling experiments, the maximum thrust force predicted by the FE model is within 1.991% for the CD854 and 8.976% for the CD856 drill. However, one must consider the entire load profile prediction and understand that there is room for improvement in the FE prediction.

**Figure 16:**
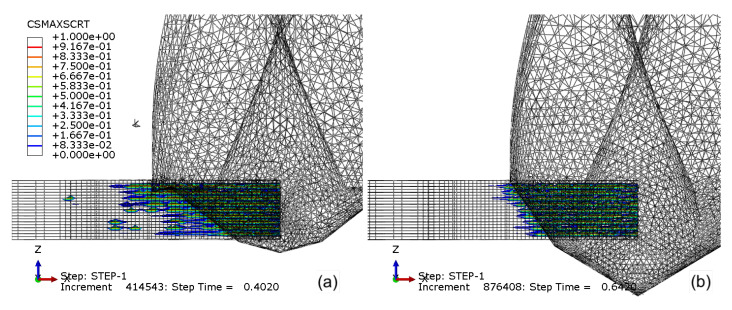
Damage initiation (CSMAXSCRT) of the cohesive surface when drilling with (**a**) CD854 and (**b**) CD856.

Figure 16a,b details the damage initiation (CSMAXSCRT) of the cohesive surface when drilling with CD854 and CD856, respectively. The maximum stress criteria output magnitude ranges from 0 to 1, with zero signifying undamaged cohesive surfaces and one identifying damage evolution initiated. When drilling with CD854, more cohesive damage is observed when compared to CD856. The reduced cohesive damage is a result of the smaller double-point angle feature found in the CD856 geometry. The smaller point angles reduce the thrust force induced into the CFRP, which lessens the bending and resulting delamination between the plies. This was observed experimentally by Ahmet et al., who experimentally studied the relationship between point angle and delamination, and point angle and thrust force through an analysis varying spindle speed and feed rate [59]. Most critically, the small point angle of 90° versus the 118° or 130° demonstrated the least delamination. The narrow point angle transforms the axial thrust force into radial compression. This transforms a portion of the axial, mode I—opening failure—to mode II—in-plane shear. More specifically, the axial thrust causing bending and delamination between the plies is reduced. This observation agrees with Su et al., who demonstrated by modifying the spur edge from axial to a double-point angle inspired spur edge that the thrust force included a radial component causing compression on the laminate hole-wall, thereby reducing delamination damage [60].

Implementing the cohesive interaction in the FEM model facilitates the ability to capture the delamination damage in the composite. This occurs at the entry and exit of the drilling in the UD-CFRP laminate and must be minimized to maintain the composites’ integrity near the hole. This is especially true for thin-walled composites. Thick composites have significant material, resulting in a greater second moment of inertia and a higher resistance to bending. However, the thick wall composite is still susceptible to delamination due to the mode I—opening—which is between plies as the tool exits the composite. Thin-walled composites are prone to mode I—opening delamination near the entry and exit due to the decreased second moment of inertia.

**Figure 17:**
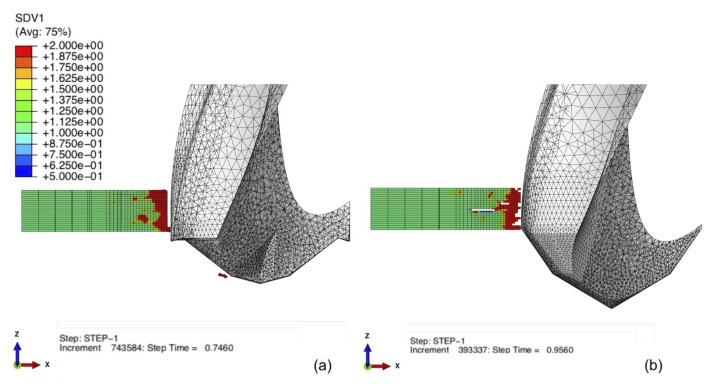
Hole quality when drilling with (**a**) CD854 and (**b**) CD856.

The FE model developed in this research also predicts hole quality with respect to geometry. Figure 17a,b details the total damage variable (SDV1) that controls element deletion. The CD854 with the candlestick geometry shown in Figure 17a demonstrated a more precisely cut hole and parallel wall. The CD856 as shown in Figure 17b demonstrated a less precisely cut wall with some damage resulting in element deletion radially into the laminate. This supports the hole quality results from experiments shown in Figure 12a,b. A precise hole being cut with the CD854 in Figure 17a and more spaling and fraying observed by the CD856 hole shown in Figure 17b. The FEA results and observations experimentally validate the functionality of the spur cutting edge of the CD854, which creates a more precise cutting path, resulting in a more accurate drilled hole geometry.

## 5. Conclusions

A macro 3D FE drilling model was presented that could be used as an accurate simulation tool to model the effects of drill geometries with reasonable computational time. Additionally, a numerical analysis was performed to investigate the influence of mass scaling, bulk viscosity, friction, strain rate strengthening, and cohesive surface modelling. The following conclusions were made based on the definite boundaries and magnitude that was used in this investigation:

- Mass scaling has a substantial effect on computational time reduction. Consideration must be made to minimize the dynamic effects caused by the increased density of the elements when implementing mass scaling.
- Linear and quadratic bulk viscosity parameters can mitigate the noise generated during the simulation of CFRP laminate drilling. Effective selection of the bulk viscosity parameters can improve thrust force prediction with a marginal increase in computational time.
- Modelling the tool as a 3D surface, versus the rigid 3D body tool commonly used in literature, demonstrated computational advantage and accuracy in model prediction.
- When cohesive surface modelling was incorporated into the 3D drilling model, the predicted thrust force signature agrees better in terms of magnitude and profile when compared with those acquired experimentally.
- The 3D drilling model could accurately predict the thrust force and hole quality generated by two different drills. The simulated results show that, with a double-angle drill tip geometry, inter-ply damage was reduced. With the “candle-stick” drill tip, the hole quality was improved.
- In comparison to the drilling experiments, the maximum thrust force predicted by the FE model is within 1.991% for CD854 and 8.976% for the CD856 drill.
- The CD854 “spur-edges” drills a higher-quality hole; however, the CD856 double-angle reduces delamination. Further investigation continues into the modification of the spur-edge to reduce inter-ply damage by promoting axial compression.

## Figures and Tables

**Table 1:** Standard Modulus (SM) Carbon Fibre-Reinforced Plastic (CFRP)—material properties.

SM UD CFRP	0	90
Longitudinal Modulus (GPa)	135	10
Transverse Modulus (GPa)	10	135
In-plane Shear Modulus (GPa)	5	5
Major Poisson’s ratio	0.3	0.3
Ultimate (Ult.) Tensile Strength (MPa)	1500	50
Ult. Comp. Strength (MPa)	1200	250
Ult. In-plane Shear Strength (MPa)	70	70
Ult. Tensile Strain	1.05	0.5
Ult. Comp. Strain	0.85	2.5
Density (g/cm${}^{3}$)	1.6	

**Table 2:** Effect of mass scaling.

Time Step (sec.)	CPU Time (days)	Complete (%)	Mass Change (%)	Net Weight (kg)
2.5 × $10^{-6}$ s	0.748	100	3.419 × $10^{3}$	5.950172
1.0 × $10^{-6}$ s	0.962	100	5.469 × $10^{2}$	1.093824
7.5 × $10^{-7}$ s	2.076	100	3.076 × $10^{2}$	0.689199
5.0 × $10^{-7}$ s	3.086	100	1.366 × $10^{2}$	0.400060
2.5 × $10^{-7}$ s	5.897	100	3.403 × $10^{1}$	0.226627
1.0 × $10^{-7}$ s	13.128 *	93.3	5.303	0.178053
7.5 × $10^{-8}$ s	19.615 *	39.4	2.910	0.174007
1.096 × $10^{-8}$ s	174.669 *	0.1	No Scaling	0.169087

* Analysis not completed.

**Table 3:** Parametric study on bulk viscosity.

Phase I		
**Bulk Viscosity Input** $b_{1},b_{2}$	**Change in Mass Scaling (%)**	**Thrust Force (N)**
0.1, 2.5	8.22	42.43
0.4, 2.5	93.1	56.01
0.7, 2.5	227	83.14
1.1, 2.5	492	139.62
**Phase II**		
**Bulk Viscosity Input** $b_{1},b_{2}$	**Change in Mass Scaling (%)**	**Thrust Force (N)**
0.04, 0.8	−3.95	31.12
0.06, 1.2	0.00	34.42
0.08, 1.6	3.95	36.61
0.10, 2.0	8.22	36.14

* Default parameters.

**Table 4:** Friction coefficient study.

Friction Coefficient	Thrust Force (N)	In-Plane Force (N)
0.05	34.76	2.32
0.15	34.65	2.43
0.35	38.61	2.29
0.55	35.81	2.62

## Data Availability

Please contact authors.

## Nomenclature

ϵ: strain
*v*: Poisson’s ratio
σ: stress (Pa)
*E*: Young’s modulus (Pa)
*G*: shear moduli (Pa)
f,m,c: fibre, matrix, composite
$\sigma_{11}$: longitudinal (fibre) stress (Pa)
$\sigma_{22},\sigma_{33}$: transverse (matrix) in-plane stress, transverse out-of-plane stress (Pa)
$\sigma_{12},\sigma_{13}$: longitudinal in-plane shear stress, longitudinal out-of-plane shear stress (Pa)
$\sigma_{23}$: transverse shear stress (Pa)
$\sigma_{eq}$: equivalent stress (Pa)
$\hat{\sigma }$: effective stress tensor (Pa)
$\sigma_{0}$: reference stress (Pa)
$\epsilon_{0}$: reference strain
$\dot{\epsilon_{0}}$: reference strain rate (s${}^{-1}$)
$u_{n}$: normal separation
$u_{t}$: tangential separation
$\delta_{n}$: maximum normal separation
$\delta_{t}$: maximum tangential separation
$T_{n}$: normal traction
$T_{t}$: tangential traction
$G_{Ic}$: mode I fracture energy (kJ/m${}^{2}$)
$G_{TIc}$: mode II fracture energy (kJ/m${}^{2}$)
$R_{a}$: average surface roughness (μm)
$V_{f}$ or $\phi_{f}$: fibre volume fraction
$V_{m}$ or $\phi_{m}$: matrix volume fraction
θ: fibre orientation (°)
ρ: density (kg/m${}^{3}$)
$L^{C}$: characteristic length
*D*: damage variable
CDM: continuum damage mechanics
CFRP: carbon fibre-reinforced polymer
CODAM: composite damage model
CZM: cohesive zone model
FD: fibre damage
FE: finite element
FRP: fibre-reinforced polymer
HM: high modulus
MD: matrix damage
RVE: representative volume element
